# Functionalization and Surface Modification of Mesoporous Hydrophobic Membranes by Oligomers and Target Additives via Environmental Crazing

**DOI:** 10.3390/membranes13050466

**Published:** 2023-04-27

**Authors:** Alena Yu. Yarysheva, Semen N. Klyamkin, Larisa M. Yarysheva, Olga V. Arzhakova

**Affiliations:** Faculty of Chemistry, Lomonosov Moscow State University, Leninskiye Gory 1/3, 119991 Moscow, Russia

**Keywords:** mesoporous membranes, hydrophilization, water vapor permeability, surface modification

## Abstract

This work offers an ecologically friendly and facile approach for the modification of high-tonnage commercial polymers, including polypropylene (PP), high-density polyethylene (HDPE), and poly(ethylene terephthalate) (PET), and preparation of nanocomposite polymeric membranes via incorporation of modifying oligomer hydrophilic additives, such as poly(ethylene glycol) (PEG), poly(propylene glycol) (PPG), polyvinyl alcohol (PVA), and salicylic acid (SA). Structural modification is accomplished via the deformation of polymers in PEG, PPG, and water-ethanol solutions of PVA and SA when mesoporous membranes are loaded with oligomers and target additives. The content of target additives in nanocomposite membranes is controlled by tensile strain, and the level of loading can achieve 35–62 wt.% for PEG and PPG; the content of PVA and SA is controlled by their concentration in the feed solution. This approach allows for the simultaneous incorporation of several additives which are shown to preserve their functional performance in the polymeric membranes and their functionalization. The porosity, morphology, and mechanical characteristics of the prepared membranes were studied. The proposed approach allows an efficient and facile strategy for the surface modification of hydrophobic mesoporous membranes: depending on the nature and content of target additives, their water contact angle can be reduced to 30–65°. Water vapor permeability, gas selectivity, antibacterial, and functional properties of the nanocomposite polymeric membranes were described.

## 1. Introduction

Membrane materials are widely used in all spheres of human life, from modern industry, water treatment, and energy to modeling of biological processes, thus providing comfortable and healthy living conditions and environmental safety. The special importance of polymeric membranes is associated with their high functional performance, mechanical strength, chemical stability, and low cost. Porous membrane materials based on polypropylene (PP), high-density polyethylene (HDPE) and polyethylene terephthalate (PET) are used for blood oxygenation, water purification, distillation and desalination, biosensing, membrane separation, textile industry, etc. [1,2,3].

Polymeric high-performance membranes are prepared by various methods and protocols, including phase inversion, high-energy irradiation (so-called nuclear filters), electrospinning, removal of inorganic or organic additives [4,5,6,7,8,9,10], etc. However, despite evident benefits, the above methods and approaches also suffer from certain limitations related to the limited choice of polymers, poor solubility, complex and multistage protocols, high consumption of toxic organic solvents and pore-forming additives, ecological hazards, etc. Porous polymeric membranes are also fabricated by stretching and controlled heat treatment when the process involves the stage of orientational drawing [11,12,13,14,15].

The performance of modern polymeric membranes is primarily governed by morphology and structural parameters of the membrane materials (porosity, pore size and pore size distribution, thickness, tortuosity, etc.) as well as by surface properties (hydrophilic lipophilic balance (HLB), adhesion, surface pattern, surface charge, etc.). For example, the high hydrophobicity of polyolefin-based membranes prevents their efficient use due to severe fouling. In this connection, the key direction of membrane science and technology is concerned with the studies on the directional design of surface and interfacial patterns. The vital problems of membrane fouling can be solved by hydrophilization, the development of steric hindrances, or the introduction of agents providing electrostatic repulsion. General approaches to hydrophilization of membranes involve plasma treatment, graft polymerization of hydrophilic polymers, dip coating or spin coating by layers or molecules capable of interactions with the membrane surface, layer-by-layer self-assembly and step-by-step deposition of functional polymers which provide electrostatic interactions or the formation of hydrogen or covalent bonds, mussel-inspired surface deposition, formation of amphiphilic surfaces with a lotus effect, etc., have been summarized in numerous reviews [16,17,18,19].

Modern commercial membranes are becoming more complex and smarter, and the efforts of researchers are focused on the development of high-performance materials with improved service properties using ecologically friendly “green” approaches. This work offers an efficient and facile approach for the preparation of mesoporous hydrophilic and filled membranes based on high-tonnage commercial polymers (PP, HDPE, PET) with improved properties via environmental crazing of polymers in PPG, PEG, and water-ethanol solutions of PVA, including those containing salicylic acid (SA). Environmental crazing is a specific mode of plastic deformation of polymers in the presence of physically active liquid environments (PALE) which is accompanied by the development of a nanoscale fibrillar-porous structure [20]. Environmental crazing proceeds so that the as-formed porous structure is continuously loaded with the PALE and the promoting effect of the PALEs on the plastic deformation of polymers is concerned with the depression of surface energy or plasticization of polymers [20,21,22]. In common practice, PALEs are organic solvents (alcohols, hydrocarbons, chlorinated and aromatic hydrocarbons), emulsions and supercritical fluids whose Hildebrand solubility parameters are close to that of polymers [20,23,24,25].

In this work, polyolefins (HDPE, PP) were selected as common representatives of the family of semicrystalline polymers which experience intercrystalline environmental crazing whereas PET is an amorphous glassy polymer that experiences classical environmental crazing. PEG and PPG as oligomers are widely used in the pharmaceutical and food industry. The use of ecologically safe hydrophilic PEG and PPG as PALEs can promote environmental crazing in “green” mode and provide surface modification and hydrophilization of hydrophobic PP, HDPE, and PET whereas low-molecular-mass and high-molecular-mass compounds are able to penetrate the mesoporous structure of polymers upon crazing [26,27,28]. This work presents the study on the potential of environmental crazing as the strategy for the preparation of multicomponent membranes materials that synergetically unite the functional properties of incorporated additives and porous host matrix: membrane performance, vapor permeability, selectivity, hydrophilicity, antibacterial and sensing properties as well high mechanical robustness. The principal objective of this work is aimed at the comprehensive research of the structure and properties of nanocomposite and supported membranes and highlighting the benefits and promises of the strategy of environmental crazing.

## 2. Materials and Methods

### 2.1. Materials

Porous polymeric host matrixes were prepared from commercial films of semicrystalline extruded lamellar HDPE (thickness 60 μm, m.m. 127 kDa, degree of crystallinity 67%); isotactic spherulitic PP after annealing at 140 °C for 3 h (thickness 30 μm, 250 kDa, degree of crystallinity 57%); amorphous unoriented PET (thickness 100 μm, m.m. 27 kDa). As a PALE for deformation and hydrophilization of polymers, PPG (Merck, Darmstadt, Germany, m.m. 1200 Da), PEG (Loba Chemie, Mumbai, India, m.m. 400 Da), water-ethanol (vol/vol = 3/2) solutions containing 3 wt.% of PVA (150 kDa, grade 16/1, the dynamic viscosity of 4% solution is 12–17 Pa-s-cP, degree of saponification 98–99%), solutions of salicylic acid (SA) in ethanol (1–20 wt.%).

### 2.2. Preparation of Nanocomposite Membranes and Their Surface Modification

Polymer films were stretched in PEG and PPG at 22–25 °C with a strain rate of 1 mm/min to different tensile strains ε; in water-ethanol solutions of PVA and SA, the strain rate was 5 mm/min. The gage size of the samples was 35 × 70 mm^2^. After stretching, the samples were carefully cleaned to remove the residual PEG and PPG from the surface. The samples were blotted with filter paper until they remained clean. For each tensile strain, at least six samples were prepared. Deviation from the average values of PEG or PPG content did not exceed 5%. To prevent the subsequent shrinkage, the samples were fixed in a circular framework and were subjected to annealing at 100 °C for PP and 110 °C for HDPE.

### 2.3. Methods

#### 2.3.1. Estimation of Volume Porosity

The mechanism of deformation porosity of the test samples was measured via changes in geometric dimensions upon stretching in the PALE to given tensile strains ε through the following equation:

W = [ΔV/V_t_] × 100, % (1)

where ΔV is the volume gain, and V_t_ is the current volume. The measurements were performed for five samples. The mean-square deviation was 3%. Theoretical porosity is estimated as:

W = ε/(1 + ε) × 100, %(2)

where ε is the relative strain.

#### 2.3.2. Low-Temperature Strain Recovery

Shrinkage of the samples (λ) was calculated as:

λ = [(L − L_t_)/(L − L_0_)] × 100, % (3)

where L is the length of the sample after deformation, L_t_ is the length of the sample after shrinkage, and L_0_ is the initial length of the sample.

#### 2.3.3. Estimation of the Composition of the Composite Membranes

The content of the incorporated additive (guest) was calculated as:

Δm/m_t_ = (m_t_ − m_0_)/m_t_ × 100, % (4)

where m_t_ is the weight of the loaded sample, m_0_ is the initial weight of the sample (before loading).

#### 2.3.4. Tensile Tests

Tensile tests of polymeric films and membranes were performed under uniaxial stretching with a constant strain rate of 1 mm/min on an Instron 4301 universal tensile machine (Instron Ltd., High Wycombe, UK). The samples were cut as dumb-bell shaped specimens with a gage size of 20 × 6 mm^2^. To study the mechanism of deformation of polymers in liquid environments, the film samples were fastened in the clamps in a transparent plastic bag. In each testing run, not less than 5–7 samples were tested. The mean square error was below 10%.

#### 2.3.5. Differential Scanning Calorimetry (DSC)

Thermophysical characteristics of the nanocomposite membranes were studied on a TA 4000 Mettler thermal analyzer (Mettler-Toledo International Inc., Columbus, OH, USA). The heating rate was 10 K/min. The weight of the test samples was 1–2 mg. The degree of crystallinity of polymers and oligomers was calculated as:

χ = [ΔH/ΔH_χ=100%_] × 100, % (5)

where ΔH is the experimental heat of fusion of polymers, ΔH_χ=100%_ is the heat of fusion of ideal crystal—190 J/g (PP), 293 J/g (HDPE).

#### 2.3.6. Atomic Force Microscopy (AFM)

The structure of mesoporous membranes was studied by scanning probe microscopy with a Multimode eight microscope equipped with a Nanoscope V controller Veeco (San Jose, CA, USA). The measurements were performed in air at room temperature in the PeakForce Tapping QNM nonresonant scanning mode. Silicon nitride cantilevers with SNL-10 single-crystalline silicon tip apexes (Bruker, Billerica, MA, USA) operating at a nominal resonance frequency of 65 kHz and a force constant of 0.35 N m^−1^ were used as the probes.

#### 2.3.7. Scanning Electron Microscopy (SEM) with X-ray Spectral Analysis

Distribution of incorporated PVA and SA in the NCM was performed using a Hitachi S-520 scanning electron microscope (Tokyo, Japan). Prior to the test, the samples were subjected to brittle fracture in liquid nitrogen and the fractured surface was decorated with gold by ionic plasma sputtering using an Eiko IB-3 setup.

#### 2.3.8. Wettability and Water Contact Angles of Membranes

The wettability of pristine polymers and NCMs was studied by a sessile drop method. Water contact angle (WCA) was measured for deionized water (4 μL) at room temperature. The shape of water droplets was analyzed using a Kruss DSA30E image analysis software (Hamburg, Germany). The measurements were performed for at least 5–7 droplets with an accuracy of ±3°.

#### 2.3.9. Water Vapor Permeability and Water Vapor Transmission Rate (WVTR)

WVTR was measured according to the Standard Test Methods ASTM E96 [29]. The test cell (25 mL) was filled with water and a relative humidity (RH) of 100% was maintained. Then, the water-loaded cell and test samples were placed in the sealed vessel containing calcium chloride to reduce the relative humidity to zero level. WVTR (K) was calculated as

K = (m_0_ − m_t_)/(S × t) (6)

where m_0_ and m_t_ stand for the initial and current weight of the cell with the specimen at time t, S is the membrane area.

#### 2.3.10. Gas Permeability Measurements

Gas permeability was measured using a GKSS automatic barometric equipment which includes a Pfeiffer Vacuum station, an MKS Baratron pressure sensor, and Labview software. The measurements were carried out for O_2_, N_2_, CO_2_, CH_4_, and H_2_ at (25 ± 1) °C. Gas flux was supplied at 1 bar, and integral registration mode was applied. Gas permeability of flat-sheet membranes was defined as the gas flux through the membrane at a given pressure difference as:

P = (J × l)/ (∆p × A)(7)

where P is the permeability of the membrane, J is the gas flux through the membrane, ∆p is the pressure gradient, l is the membrane thickness and A is the membrane working area. The ideal selectivity of the membranes, α, was calculated as the ratio of permeability of individual gases.

#### 2.3.11. Assessment of Antibacterial Activity of Nanocomposite Membranes

The antimicrobial activity of the NCMs was studied by the standard procedure [30]. Bovine serum albumin (BSA) in agar was used as a nutrient medium. The antimicrobial activity of the samples was studied using the strains of *Escherichia coli*. The Petri dishes were inoculated with a standardized inoculum of test microorganisms. The samples together with the blank samples were placed into the Petri dishes. The strain was grown in BSA/agar at 37 °C and allowed to stay for 24 h. Antimicrobial activity was estimated by the size of inhibition zones where the strain growth ceased.

## 3. Results and Discussion

### 3.1. Environmental Crazing of PP, HDPE, and PET

Supported membranes based on PP, HDPE and PET with oligomers PEG and PPG as well as nanocomposite HDPE-PVA, HDPE-SA, and HDPE-PVA-SA membranes (NCMs) were prepared using the strategy of environmental crazing when polymer films were stretched in PPG, PEG, and water-ethanol solutions of PVA and SA. Noteworthy is that the above PALEs do not induce the swelling of polymers. In contrast to typical volatile PALEs, PEG and PPG oligomers are characterized by higher viscosity. For example, the viscosity of low homologs of aliphatic alcohols or hydrocarbons does not exceed 10 cP whereas the viscosity of PPG 1200 is 270 cP at 20 °C. The effect of the PALE on the mechanism of deformation of polymers is assessed by tensile tests and measurements of porosity upon stretching [20].

**Figure 1:**
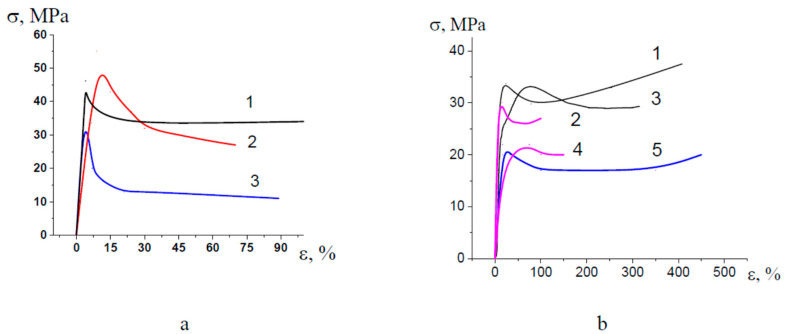
Stress-strain curves (initial regions) illustrating tensile drawing of (**a**) PET (1) in air, (2) PPG, (3) PEG; (**b**) PP (1) in air, (2) PEG; HDPE (3) in air, (4) PEG, (5) water-ethanol solution containing PVA and SA (concentration 3 and 1 wt.%, respectively).

Figure 1 shows the stress-strain curves describing the deformation of polymer films in air and in PEG and PPG. The deformation of amorphous glassy PET in PEG proceeds at a lower stress level (by 1/3) as compared with that in the air (Figure 1a, curves 1, 3); for semicrystalline PP, no marked changes in the corresponding stress-strain curves are observed, and yield stress decreases only by 5 MPa (Figure 1b, curves 1, 2). For HDPE upon deformation in PEG, yield stress decreases by a factor of 1.5 as compared with that upon deformation in air (Figure 1b, curves 3, 4). HDPE shows a similar behavior upon deformation in the water-ethanol solutions containing PVA and SA (Figure 1b, curve 5).

On passing to a more viscous PPG, yield stress PET increases up to the stress level as observed upon stretching in air (45–50 MPa), and this fact can be explained by hindered penetration of a viscous fluid to a tip of a growing craze; however, craze tip advance in PET provides a quick stress drop immediately after the yield point without the region of steady-state deformation (Figure 1a, curve 2).

Amorphous PET and semicrystalline PP and HDPE have different supramolecular structures and, as a consequence, experience different mechanisms of environmental crazing: classical crazing for PET and intercrystalline crazing for PP and HDPE [24,25]. However, their behavior upon deformation in the PALEs under study appears to be similar (reduced tensile stress as compared with that in air).

This evidence allows the conclusion that the selected PALEs show a high crazing-promoting efficiency for polymers due to the depression in their surface energy (the Rehbinder effect in polymers) [20,31] and/or plasticization due to stress-induced swelling [32].

### 3.2. Structure of Membranes

**Figure 2:**
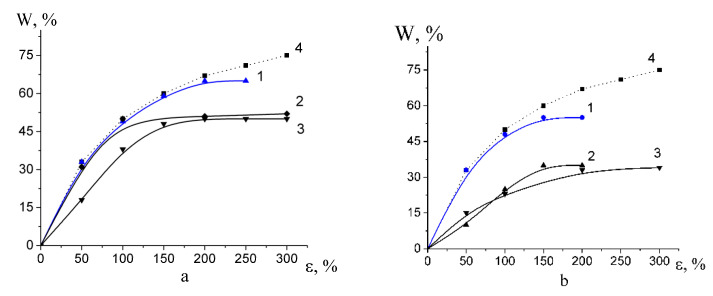
Volume porosity versus tensile strain (**a**) for (1) PET in PPG; (2) PET in PEG and (3) PP in PEG; (4) theoretical curve for “ideal” crazing; (**b**) for (1) HDPE in PPG; (2) in PEG and (3) in water-ethanol solution; (4) theoretical curve for “ideal” crazing.

Deformation of polyolefin and PET films in the PALEs is accompanied by the development of porosity when volume strain increases as the tensile strain increases up to ~100%; at higher tensile strains, the porosity-(tensile strain) curves smoothly increase or level off (Figure 2). As compared with theoretical curves (curves 4 in Figure 2) for “ideal” crazing, experimental curves upon deformation in a more viscous PPG (curves 1 in Figure 2) are similar to theoretical curves and this evidence supports the conclusion concerning the stabilizing role of a highly viscous fluid in the development of mesoporous structure via environmental crazing. The values of porosity of HDPE in the water-ethanol solutions as a typical PALE and in PEG are similar but much lower than theoretical values (Figure 2b, curve 2, 3). Maximal porosity achieved upon deformation in PEG is the following: 35 (HDPE), 48 (PET, PP) vol.%; in PPG—50 (HDPE), 60 (PP, PET) vol.%, respectively.

Classical crazing (PET) and intercrystalline crazing of PP and HDPE are known to provide the development of a high surface area (up to 100 m^2^/g) due to self-induced nanodispersion of the polymer [20]. As the tensile strain increases, pore size and craze opening (fibril length) tend to increase. The as-formed fibrillar-porous structure of crazes presents an unstable colloidal system composed of nanoscale aggregates (craze fibrils), which tends to reduce its surface energy due to coagulation of fibrils by lateral surfaces, thus leading to the deviation of the experimental porosity from the “ideal” case. Upon stress relaxation, this thermodynamically unstable system experiences even more dramatic structural rearrangements and low-temperature spontaneous strain recovery [20]. This process is accompanied by so-called syneresis which involves the release of a liquid “encaptured” within the porous structure upon crazing.

**Figure 3:**
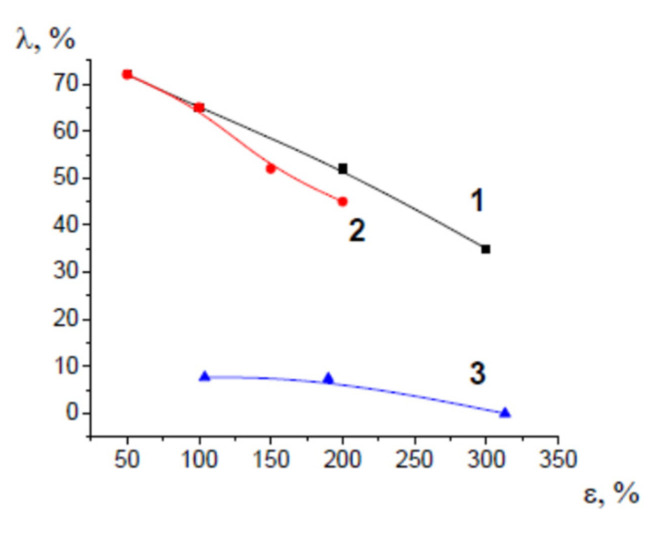
Strain recovery versus tensile strain for (1) HDPE, (2) PP, and (3) PET after their deformation via environmental crazing.

Figure 3 shows the strain recovery plotted against tensile strain. Strain recovery decreases with increasing the tensile strain because, at high tensile strains ε, irreversible changes (lamella fragmentation, shearing, sliding of lamellar fragments) for semicrystalline polymers. Noteworthy is that amorphous glassy PET experiences crystallization upon the deformation process, and its degree of crystallinity is 23%. Strain recovery of PET appears to be much lower than those of HDPE and PP and does not exceed 8% for all tensile strains.

To prevent strain recovery and shrinkage, the deformed films with fixed dimensions were annealed under isometric conditions. Annealing is accompanied by additional crystallization of semicrystalline PP, and HDPE. Under the selected annealing conditions, the degree of crystallinity of PP and HDPE increases by 3–6%. Evidently, temperature-induced crystallization provides stabilization of the porous structure of polymers after environmental crazing. The PET membranes containing highly viscous PEG and PPG appear to preserve their stability even without annealing. After annealing, PP and HDPE strain recovery proceeds for about 30 days, but this value is below 5–15% for all tensile strains. Shrinkage is accompanied by the “blooming” of the incorporated oligomers, and their content in the composite decreases. The net release of the additives upon strain recovery depends on the tensile strain upon environmental crazing of PP and HDPE: by 3–6 wt.% (PPG) and by 10–23 wt.% (PEG) with respect to their initial content in the composite. Then, all samples were examined within 1–3 months when the equilibrium state was attained.

**Figure 4:**
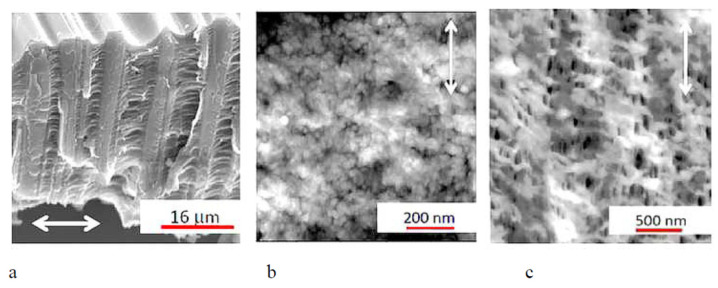
(**a**) SEM micrograph of the fractured surface of the PET membrane after classical environmental crazing and (**b**) AFM images of PP and (**c**) HDPE membranes after intercrystalline environmental crazing and annealing. Direction of stretching is denoted by arrow.

The difference between porous PET membranes after classical environmental crazing and semicrystalline PP and HDPE after intercrystalline environmental crazing can be explained by the analysis of SEM and AFM images of the corresponding membranes (Figure 4). In the case of the PET membranes, the structure is composed of alternating crazes with their fibrillar-porous structure and regions of bulk unoriented polymer. Both craze walls and a highly viscous fluid within the pores “hold” the porous structure and prevent strain recovery of the PET membranes upon stress relaxation (Figure 4a). For semicrystalline spherulitic PP, the fragments of crystalline lamellae are visible, but the pores are not uniquely defined (Figure 4b). Deformation of semicrystalline HDPE films with their row-nucleated structure proceeds via the separation of crystalline lamellae which is accompanied by fibrillation and the development of porosity within interlamellar amorphous regions. In this case, all pores are slit-shaped and elongated in the direction of the tensile drawing (Figure 4c).

According to the data of low-temperature nitrogen adsorption, the pore dimensions of the porous membranes lie within a broad range from several to hundreds of nanometers: average pore size is 6 nm for PP, 28 nm for HDPE at ε = 200%, and 11 nm for PET at ε = 100%. Hence, according to the IUPAC classification, the membranes under study belong to the class of mesoporous materials [33].

**Figure 5:**
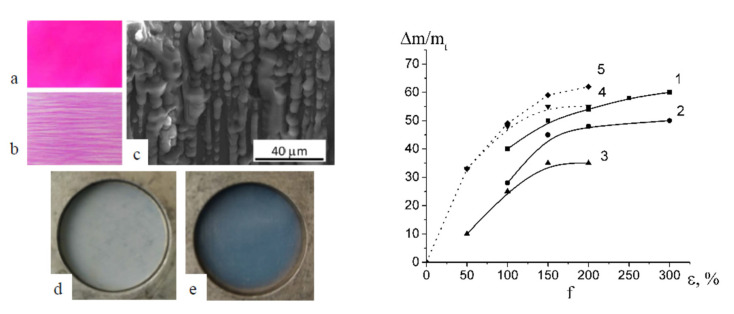
Micrographs of the (**a**) PP-PEG, (**b**) PET-PEG samples after dye-contrasting tests (Rhodamine 6G as a colorant); (**c**) SEM image of the fractured surface of the PET-PEG membrane, (**d**) HDPE membrane; (**e**) HDPE-PEG membrane; (**f**) content of PEG in (1) PET, (2) PP, (3) HDPE membranes; content of PPG in (4) HDPE, (5) PET.

When PEG containing dye Rhodamine 6G is introduced into the porous structure of porous membranes under study, the PP membrane is seen to be uniformly pink colored upon intercrystalline environmental crazing (Figure 5a) and this uniform coloring is provided by the uniform distribution of nanoscale pores. The incorporation of dye-containing PEG into PET makes it possible to stain and reveal the crazed regions alternating with uncolored regions of bulk undeformed polymer (Figure 5b). The SEM microphotographs of the PET membranes after their deformation in PEG via classical environmental crazing show that, under vacuum, PEG oozes out from the membrane (Figure 5c) in full accordance with the distribution of pores within crazes (Figure 4a). Thus, the deformation of polyolefins and PET in common PALEs and in highly viscous fluids proceeds via environmental crazing and the formation of a mesoscale fibrillar-porous structure. In the case of classical crazing, the structure of the membranes contains alternating porous crazes and regions of the bulk polymer as craze walls; for environmental crazing of semicrystalline polymers, the porous structure is localized within interlamellar regions.

### 3.3. Liquid Supported Nanocomposite Membranes Containing PPG and PEG

Environmental crazing of polymers provides a gradual loading of the PALE into the as-formed porous structure under the action of negative hydrostatic pressure. Figure 5d shows that the pristine unfilled porous HDPE membrane strongly scatters light due to the surface light-scattering pattern of the membranes, whereas the PEG-loaded HDPE membrane is transparent (Figure 5e).

This approach allows for the one-stage preparation of PET-based nanocomposite membranes containing a hydrophilic additive. In the case of PP and HDPE, this result is achieved in two stages: stretching/loading and annealing of the resultant membranes. Figure 5f shows the PPG and PEG content in the loaded membranes plotted against the tensile strain of the host matrixes. The content of oligomers in the supported membranes increases with overall porosity (Figure 2). Since the additive is accommodated within nanoscale pores, its aggregation is prevented by space confinement (pore walls, fibrils). Hence, the resultant materials can be classified as liquid-supported membranes and nanocomposite membrane materials (NCM). The maximum content of PEG was 60 wt.% in the PET-based membranes, 50 wt.% in PP, and 35 wt.% in HDPE, and PPG—54 wt.% in HDPE and 62 wt.% in PET.

### 3.4. Nanocomposite Open-Porous HDPE-Based Membranes Containing PVA and Salicylic Acid

The principal benefits of environmental crazing for the preparation of porous polymeric materials are concerned with the possibility of the introduction of both low-molecular-mass and high-molecular-mass functional additives to the porous structure of polymers. This work offers a facile approach allowing for the preparation of HDPE-based NCM containing PVA as a high-molecular hydrophilic additive and salicylic acid as an antibacterial agent. The above additives (SA, PVA) can be embedded into the HDPE host-matrix in one stage when the polymer is stretched in SA and PVA-containing solution.

**Figure 6:**
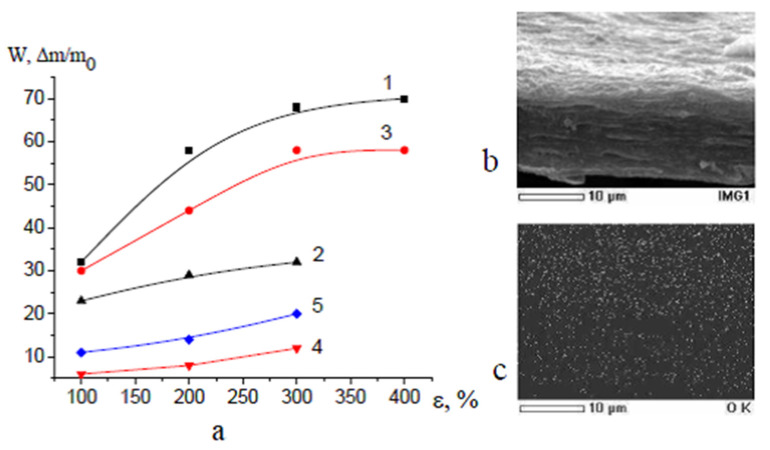
(**a**) Volume porosity (1,2) of NCM and content of (3,4) SA and (5) PVA in NCM prepared (3) by stretching of HDPE in 20 wt.% solutions containing SA and doped with (4) SA and (5) PVA solutions; (**b**) SEM image of the fractured surface of the stable HDPE-based membrane containing 14 wt.% of SA; (**c**) distribution of oxygen atoms on the fractured surface.

The content of incorporated PVA and SA in the NCM is controlled by the tensile strain of the host matrix and the concentration of the functional additive in the feed solution. The removal of a volatile filler water-ethanol solution from the membrane and subsequent annealing under isometric conditions preserves the open porosity of the NCM when additives are loaded from low-concentration solutions (1–10 wt.%). Figure 6 shows that the SA additive is uniformly distributed within the HDPE host matrix as evidenced by the homogeneous distribution of oxygen atoms throughout the whole volume.

Surface functionalization of the membranes can be accomplished by surface modification of open-porous HDPE membranes with functional additives and this efficient and low-cost approach allows the preparation of task-oriented membrane materials. For example, the incorporation of an antibacterial agent (SA) and hydrophilic agent (PVA) makes it possible to prepare membranes with antibacterial and hydrophilic properties. Therefore, the strategy of environmental crazing offers the protocol for the controlled design of NCMs with high content of hydrophilic oligomers (up to 60 wt.%) and multicomponent NCMs whose composition can be controlled by the tensile strain of the polymer matrix and concentration of the feed solution.

**Figure 7:**
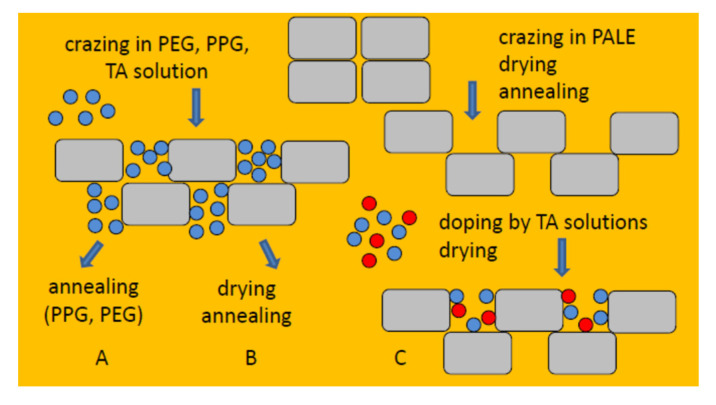
Scheme illustrating the protocol of preparation of NCM via environmental crazing. The possibility of introducing different additives is highlighted in color.

Figure 7 shows the general scheme illustrating the proposed approach for the preparation of NCMs: (A) environmental crazing of polymer films (PET, PP, HDPE) in oligomers + annealing under isometric conditions (PP, HDPE) = membranes PET, PP, HDPE with PEG and PPG; (B) environmental crazing of polymer films in the solutions containing modifying target additives (TA) in PALE + removal of a volatile liquid + annealing = HDPE-PVA, HDPE-SA, HDPE-PVA-SA membranes; (C) environmental crazing of polymer films in PALE + removal of PALE + annealing + doping with TA solutions + removal of PALE = surface-modified HDPE membranes.

### 3.5. Performance of PP, HDPE, and PET Membranes with Hydrophilic Oligomers

#### 3.5.1. Water Vapor Permeability and Wettability

The development of the surface pattern of the membranes offers an efficient route for surface modification of hydrophobic membranes according to the Cassier–Baxter heterogeneous wetting mode or mixed wetting mode [34]. In this connection, environmental crazing offers a facile approach for the development of surface patterns and modification of surface properties of membrane materials. For example, the water contact wetting angle (WCA) of initial PP and HDPE is equal to 98°; porous membranes based on HDPE and PP appear to be more hydrophobic (WCA = 105°) due to the surface pattern produced by lamellar protrusions and pore depressions. Initial PET is known to be less hydrophobic (WCA = 83°). However, the PET membrane after environmental crazing shows the wetting anisotropy: WCA along the direction of stretching WCA_↔_ = 94°, and WCA in the perpendicular direction is WCA_↕_ = 72°. This anisotropy can be explained by the formation of a highly anisotropic structure upon classical environmental crazing (Figure 4a).

Table 1 presents the summarized data on vapor permeability and wettability of the PP, HDPE and PET-based membranes containing PPG and PEG oligomers. Deformation of PET in PPG and PEG is accompanied by the development of numerous crazes, and surface anisotropy and wettability of the PET-PEG membrane is less pronounced as compared with that of PET upon environmental crazing in conventional volatile PALEs. According to the data listed in Table 1, PPG and PEG provide hydrophilization of PP, HDPE, and PET-based membranes. Noteworthy is that PPG appears to serve as a stronger hydrophilizing agent as compared with PEG: WCA of the PPG-containing membranes is equal to 38_↔_°/27_↕_° (PET), 50° (HDPE) and 45° (PP), and this value is by 20° lower than that of the PEG-containing membranes. However, the WVTR of the membranes containing less viscous PEG is shown to be ~3 (PET) and 5 times higher (PP) as compared with the corresponding values of the PPG-containing membranes. The NCM membranes appear to show high water vapor permeability (WVTR, water vapor transmission rate) 1264–1660 g/(m^2^ day)which is comparable to that of commercial analogs [35,36].

The loaded membranes do not swell in water vapors, and this fact implies that even though hydrophilic PEG and PPG are able to sorb water at relative humidity RH = 85%, these compounds being immobilized in nanopores have negligible sorption capacity due to steric hindrances. However, the high vapor permeability of the membranes under study suggests that PEG and PPG provide interconnected hydrophilic channels within the network of interconnected pores, and the diffusion flow of water vapors proceeds through these channels.

The stability of the PP-PEG and PET-PEG samples in the presence of an aqueous environment was studied. Within 24 h, half of the incorporated PEG is released from the membrane to water. Evidently, only oligomers located in the surface layers can be dissolved in water. Once PEG is released from the surface layer, the channels open for water penetration into hydrophobic mesoporous membranes appear to be closed, and the residual content of PEG in the membranes remains unchanged upon prolonged (10 days exposure to water and even upon boiling). Vapor permeability of the membranes after the partial release of PEG slightly increases, for example, WVTR PP-PEG increases from 1254 to 1356 g/(m^2^ day) when the content of PEG was reduced from 47 down to 27 wt.%. Due to annealing, the membranes preserve their stability and porosity after the partial release of PEG.

#### 3.5.2. Mechanical Properties

**Figure 8:**
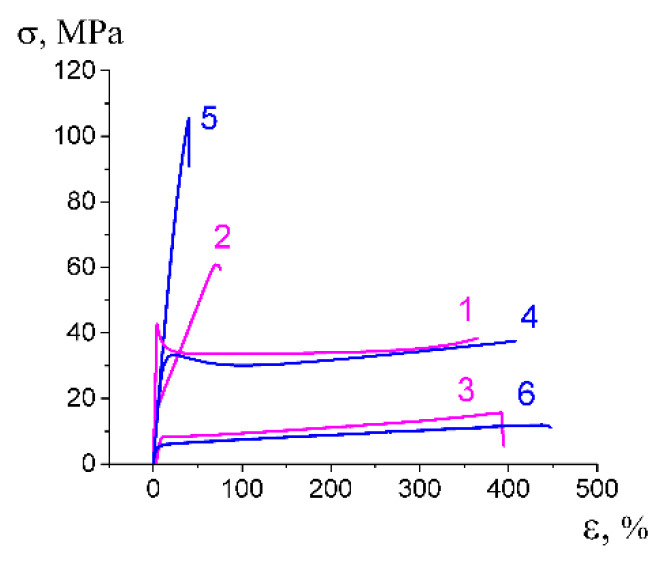
Stress-strain curves illustrating mechanical characteristics of the composite membranes: (1) initial PET; (2,3) PET (ε = 100%)-PEG (2) along and (3) perpendicular to the direction of tensile drawing; (4) initial PP; (5,6) PP (ε = 350 %)-PEG (5) along and (6) perpendicular to the direction of tensile drawing.

Mechanical tests show that the nanocomposite membranes preserve good mechanical properties when the tests are performed along the direction of tensile drawing upon environmental crazing (Figure 8). When the secondary tensile drawing is performed in the perpendicular direction, the mechanical performance appears to be even better. For PET-based (ε = 100%) and PP-based membranes (ε = 350%), the membranes are characterized by high elongation at the break—400% for PET-PEG and 450% for PP-PEG, a low elastic modulus of 50 MPa for PET-PEG NCM and 180 MPa for PP-PEG NCM. This evidence suggests that the NCM based on crazed polymeric matrixes are characterized by anisotropic structure and, as a result, anisotropic mechanical characteristics. Therefore, the proposed approach allows for the controlled design of robust nanocomposite membranes with high mechanical properties, and this issue is important for further practical applications of advanced materials.

#### 3.5.3. Gas Permeability of Liquid-Supported PP-PEG Membranes

In the modern world, ecological issues related to the accumulation of greenhouse gases, including carbon dioxide, present a huge environmental challenge from the viewpoint of the hazards of pollution and global warming. In this connection, studies on gas separation (CO_2_/CH_4_, CO_2_/N_2_ and CO_2_/H_2_) are central problems for membrane science. In recent years, polyethylene oxide (PEO) membranes have attracted special attention [37]. PEO-based membranes show their high affinity towards CO_2_; however, a high degree of crystallinity and poor mechanical strength prevent their practical use. In this work, the gas permeability of robust polymeric PP membranes (ε = 200%) containing PEG was studied (Table 2).

Maximum gas permeability of the PP-PEG membranes was observed for carbon dioxide: 11.3 barrer; the lowest gas permeability (0.5 barrer)—for nitrogen. Hence, gas selectivity (ratio of permeability coefficients of two gases) α for the CO_2_/N_2_ pair is 22.6; for CO_2_/CH_4_, gas selectivity α = 8.7. As a result, this approach offers a new route for the preparation of selective membranes for gas separation, and the benefits of this flexible approach are related to the possibility to control porosity, pore dimensions, and morphology of the membranes upon environmental crazing and post-treatment (annealing, strain recovery, rolling). This approach also offers unique advantages for the introduction of several different additives in one run or in step-by-step mode.

### 3.6. Performance of Nanocomposite HDPE-PVA, HDPE-SA, HDPE-PVA-SA Membranes

#### 3.6.1. Water Vapor Permeability and Wettability

**Figure 9:**
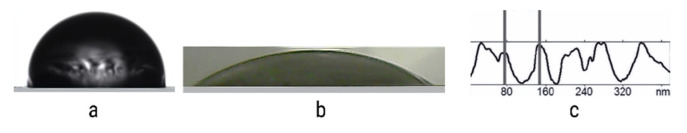
Water droplet on the surface of (**a**) HDPE film and (**b**) HDPE-PVA nanocompsite membrane, and (**c**) surface profile of the HDPE membrane after environmental crazing (ε = 200%) and annealing.

Multicomponent HDPE-SA and HDPE-PVA membranes doped with PVA and SA are permeable to water vapors: water vapor permeability of the HDPE-SA membranes is 216 g/(m^2^ day) for HDPE (ε = 100%), 850 g/(m^2^ day) (200%), HDPE-PVA 226 and 856 g/(m^2^ day) for HDPE (ε = 100 and 200%, respectively). PVA in nanoporous membranes does not swell in water, and the weight of the samples in aqueous solutions remains unchanged. The water contact angle WCA of the HDPE-PVA membrane (PVA 14 wt.%) is 30°; in other words, the incorporation of PVA into the mesoporous HDPE membrane provides its hydrophilization (Figure 9a,b), and WCA appears to be lower than the expected WCA of pristine PVA (40–45°). This evidence can be explained by the development of the surface relief as proved by profilograms constructed from the analysis of the corresponding AFM images: these profilograms clearly show nanoscale relief as ridges and depressions (Figure 9c).

#### 3.6.2. Antibacterial Properties

**Figure 10:**
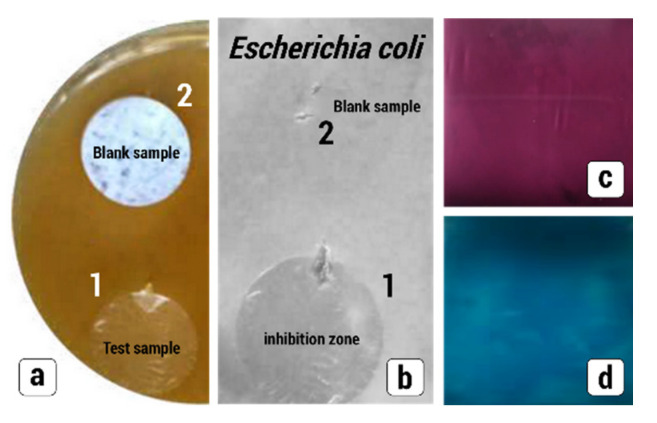
(**a**) Antibacterial tests for (1) HDPE-SA and (2) HDPE (blank sample) in the Petri dish containing Gram-negative Escherichia coli; (**b**) snapshots after the removal of the samples (24 h); (**c**,**d**) response of the nanocomposite membranes to analytes: (**c**) HDPE-SA and iron chloride (III), (**d**) HDPE-PVA and iodine.

The advantages of the functionalized NCMs are also related to the possibility to broaden the scope of target additives and to prepare diverse multifunctional membrane materials with task-oriented properties. Of special importance are the membrane materials with antimicrobial performance. In this connection, NCMs containing SA as an antibacterial agent were prepared. SA is widely used in medicine and cosmetology to treat skin disorder problems and shows a well-pronounced antiseptic and antibacterial activity towards a broad range of pathogenic organisms [38]. Antimicrobial activity of the SA-containing NCMs was studied with respect to Gram-negative bacteria (*Escherichia coli*). As follows from Figure 10a,b, the HDPE-SA membranes show a well-pronounced antibacterial activity as big-sized inhibition zones where the bacterial growth is suppressed whereas the blank sample (pristine HDPE membrane) remains inactive. The long-term antibacterial tests show that the sample preserve their antibacterial activity for several weeks.

#### 3.6.3. Sensing Characteristics

In this work, sensing characteristics of the nanocomposite membranes were studied when SA and PVA act as sensors with respect to analytes: iron ion (III) for SA and iodine for PVA. As follows from Figure 10c,d, membranes are uniformly colored by iron chloride FeCl_3_ (purple HDPE-SA) and iodine I_2_ (blue HDPE-PVA). This fact implies that the incorporated additives preserve their functional properties and can serve as sensors, and the resultant nanocomposite membranes can be used for the detection of analytes.

#### 3.6.4. Mechanical Properties

**Figure 11:**
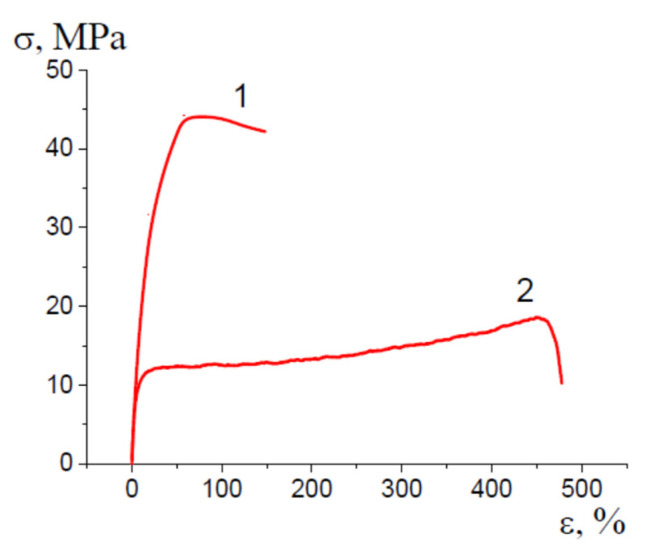
Stress-strain curve illustrating the mechanical performance of HDPE-PVA-SA NCM upon stretching (1) along and (2) perpendicular to the direction of primary tensile drawing upon environmental crazing.

The mechanical tests show that the proposed NCMs are characterized by good mechanical characteristics even though some properties (elongation at break) are slightly compromised when the stretching of the NCM is performed along the direction of tensile drawing upon environmental crazing. The mechanical performance of the HDPE-based NCM (ε = 200%) are the following: elastic modulus is 240–270 MPa, elongation at break is 450 and 150%, the yield stress is 12 and 45 MPa in perpendicular and parallel directions, respectively (Figure 11).

Therefore, the proposed approach allows for the preparation of the nanocomposite HDPE-based membranes doped with PVA and SA (HDPE-PVA, HDPE-SA, HDPE-PVA-SA) with high mechanical properties, improved wettability and vapor permeability, and new functional properties, including sensing and antibacterial activity.

## 4. Conclusions

This work offers and justifies a new facile approach for surface modification and functionalization of hydrophobic polymer materials based on high-tonnage commercial polymers (HDPE, PP, PET) via environmental crazing and the preparation of functionalized nanocomposite membranes with new task-oriented properties. Advanced membrane materials are characterized by controlled wettability, high vapor permeability, gas selectivity, and high mechanical properties. This approach offers evident advantages for the incorporation of target low-molecular-mass and oligomer additives to mesoporous host matrixes and provides their uniform and homogeneous distribution within the whole volume of the material. The benefits of this strategy of functionalization and surface modification are also concerned with the introduction of a broad range of functional additives, including agents with sensing properties and antibacterial activity. Noteworthy is that this approach shows universal character and can be applied to conventional polymers (HDPE, PP, PET) which occupy the top positions from the viewpoint of their worldwide production. Among the extra benefits, we should mention low cost, ecological safety, and technological simplicity as this approach can be implemented in a continuous operational mode using conventional industrial equipment for the orientational drawing of polymers without the use of toxic organic reagents. The flexible strategy of environmental crazing allows the controlled structural design of the membrane materials, and the controlling tools are the conditions of tensile drawing (tensile strain, strain rate, the morphology of polymers, spot crazing, etc.), the composition of the feed solutions, conditions of impregnation/doping of additives (electrolytes, antibacterial agents, medical agents, colorants, drugs, sensors, etc.), and post-treatment. This rich and flexible methodology is schematically illustrated in Figure 7. The designed functionalized membrane materials can be used as selective gas permeation membranes, ‘breathable” membranes for vapor permeability, comfort membrane materials for apparel, and membranes with antibacterial and sensing activity.

## Figures and Tables

**Table 1:** Contact wetting angle (WCA) and water vapor transmission rate (WVTR) of initial polymers and mesoporous membranes based on PP, PET and HDPE with hydrophilic oligomers.

Polymer/Additive	WCA, °	WVTR, g/(m^2^ day)
PP	98	0
PP-PEG	65	1254
PP-PPG	45	249
PET	83	0
PET-PEG	57	1660
PET-PPG	38_↔_/27_↕_	604
HDPE	98	0
HDPE-PPG	50	302

**Table 2:** Gas permeability and selectivity of PP-PEG membranes.

Permeability P, Barrer					
H_2_	CO_2_	CH_4_	N_2_	Ar	He
7	11.3	1.3	0.5	1.7	7.2
Selectivity, α					
H_2_/CO_2_		H_2_/CH_4_	H_2_/N_2_	H_2_/Ar	H_2_/He
0.6		5.4	14.0	4.1	1.0
CO_2_/H_2_		CO_2_/CH_4_	CO_2_/N_2_	CO_2_/Ar	CO_2_/He
1.6		8.7	22.6	6.6	1.6

## Data Availability

The data presented in this study are available on request from the corresponding author.
